# Twelve-Week Internet-Based Individualized Exercise Program in Adults With Systemic Lupus Erythematosus: Protocol for a Randomized Controlled Trial

**DOI:** 10.2196/18291

**Published:** 2020-11-03

**Authors:** Simone Cosima Boedecker, Keito Finn Akanby Philippi, Elmo Neuberger, Sebastian Schmidt, Daniel Pfirrmann, Nils Haller, Andreas Schwarting, Perikles Simon, Julia Weinmann-Menke

**Affiliations:** 1 Department of Rheumatology and Nephrology University Medical Center Mainz Mainz Germany; 2 Department of Sports Medicine, Disease Prevention and Rehabilitation Johannes Gutenberg University Mainz Mainz Germany

**Keywords:** systemic lupus erythematosus, physical activity, internet-based exercise program, disease activity, fatigue

## Abstract

**Background:**

Systemic lupus erythematosus is a systemic autoimmune disease, which is associated with high cardiovascular risk, a predisposition to metabolic disorders, muscle wasting, and fatigue. Exercise therapy has become an important part of the long-term treatment of comorbidities in systemic lupus erythematosus. Exercise can lead to various benefits in patients with systemic lupus erythematosus such as increased aerobic capacity and exercise tolerance, resulting in an increased quality of life, decreased depression, and decreased fatigue. At the moment, no evidence-based treatment guidelines that recommend exercise for patients with systemic lupus erythematosus exist. Also, the efficacy of different training programs requires further investigation.

**Objective:**

This study focuses on the feasibility, efficacy, and safety of an internet-based exercise program in patients with systemic lupus erythematosus. Furthermore, we investigate the feasibility and efficiency of anaerobic training compared to aerobic training.

**Methods:**

Overall, patients with systemic lupus erythematosus from the Division of Nephrology, Rheumatology, and Immunology outpatient clinic of the University Medical Center Mainz who are clinically stable status are included and randomized in an aerobic exercise group (n=10), anaerobic exercise group (n=10), or treatment as usual group (n=10). After completing initial clinical testing and physical fitness tests, patients undergo supervised 12-week online exercise programs, receiving weekly individualized training plans adapted to their physical performance. The primary outcome is change in physical fitness (VO2 peak) after 12 weeks compared to baseline. Secondary outcomes are disease activity measured via laboratory results (complement, autoantibodies) and questionnaires, as well as changes in muscle mass (anaerobic exercise group), results of the Chair-Stand test, and measurements of circulating cell-free DNA and extracellular vesicles.

**Results:**

The study was registered in May 2019. Enrollment began in May 2019. Of 40 patients who were initially screened, 30 patients fulfilled the inclusion criteria and were included in the study; 1 participant withdrew prior to the start of the exercise program. Among the 25 patients who completed the study, no serious adverse events have been reported; 3 participants withdrew during the program (due to frequent colds, n=1; Crohn relapse, n=1; physical strain, n=1), and 1 participant has not yet completed the program. Data analysis is ongoing, and results are expected to be submitted for publication in January 2021.

**Conclusions:**

We expect the online exercise intervention to be a feasible and efficient tool to provide regular individualized exercise for patients with systemic lupus erythematosus.

**Trial Registration:**

ClinicalTrials.gov NCT03942718; http://clinicaltrials.gov/ct2/show/NCT03942718.

**International Registered Report Identifier (IRRID):**

DERR1-10.2196/18291

## Introduction

Systemic lupus erythematosus is a chronic autoimmune disease that affects organs and tissues, such as skin, kidney, joints, lungs, and the central nervous system [1]. Its etiology is still unknown. Remarkably, nearly 90% of patients with systemic lupus erythematosus are females [2]. Its incidence in Germany in 2002 was 15.4 per 100,000 in males and 55.4 per 100,000 in females [3]. Worldwide, prevalence ranges from 20 to 70 per 100,000 [4]. Both geographical and racial differences seem to influence the prevalence of systemic lupus erythematosus [4].

Therapeutic approaches aim to ensure long-term survival by keeping disease activity low, reducing drug toxicity, and improving quality of life [5-7]. Due to improved drug therapies, the 10-year survival rate of patients with systemic lupus erythematosus increased remarkably from approximately 50% in the 1950s to >90% since 1990; however, long-term use of drugs and the inflammatory potential of the disease itself cause a host of comorbidities such as cardiovascular disease, end-stage renal failure, or osteoporosis [8]. Compared to the general population, the risk of death is still greater by approximately 5-fold [9,10]. It has been shown that cardiovascular disease is the main risk factor of increased death and organ damage [11].

In addition to classical therapy options, nonpharmacological interventions, which are well tolerated by the patient, are desirable. Between 67% and 90% of patients with systemic lupus erythematosus, depending on their ethnicity, report fatigue which leads to tiredness, inactivity, and thus, to a reduction of physical fitness [12,13]. This often accompanies a progressive reduction of muscle mass leading to sarcopenia [14]. Therefore, the effect of exercise in patients with systemic lupus erythematosus was investigated in several small case studies [14-20], and it was shown that exercise may be a promising augment treatment to counteract the negative effects caused by inactivity in patients with systemic lupus erythematosus (ie, fatigue, depression, disease activity, sarcopenia, and reduced aerobic capacity). Several studies have shown that physical exercise is well tolerated in systemic lupus erythematosus [21]. Cycling, running, and walking were shown to be effective and well tolerated in patients with systemic lupus erythematosus [14,18,21,22], while progressive resistance training with elastic bands is considered to be a safe method to improve muscle strength [23]; however, cardiovascular training seems to have a better effect on the quality of life of patients than resistance training does [24].

While previous studies [19,25,26] applied a supervised face-to-face exercise concept, which is very costly and time consuming due to a high staff load, more time- and cost-effective interventions are necessary to make this training concept accessible to as many patients as possible. Internet-based exercise interventions fulfill these requirements. Furthermore, individualized internet-based exercise programs, unlike group interventions, allow adjustments for each patient. There is also the possibility of a higher level of adherence, since patients are free to decide on which day and at what time they schedule their sports program, regardless of their different daily routines. Therefore, we developed an individualized internet-based exercise program. We hypothesized that patients will adhere to a 12-week exercise program and that the program will lead to a significant improvement in peak oxygen uptake, demonstrating an improvement in aerobic capacity, as well as a reduction of fatigue, depression, disease activity, and sarcopenia in patients with systemic lupus erythematosus.

## Methods

### Ethics

This study was approved by the ethics commission of the University Medical Center Mainz, Germany and the Medical Associations Rhineland-Palatinate (number 2018-13039) and conformed to the standards of the Declaration of Helsinki of the World Medical Association. Written consent was obtained from all participants at the beginning of the study.

### Recruitment

Participants aged 18-65 years and diagnosed with systemic lupus erythematosus according to the 1982 American College of Rheumatology classification criteria and the new 2019 European League Against Rheumatism/American College of Rheumatology Classification Criteria were recruited [5,27]. Detailed inclusion and exclusion criteria are listed in Table 1.

**Table 1:** Inclusion and exclusion criteria.

Type	Criteria
Inclusion	Diagnosis of systemic lupus erythematosus by the classification criteria ACR^a^ and the 2019 EULAR^b^/ACR Classification Criteria for systemic lupus erythematosus Positive antinuclear antibody titer (≥1:80) or anti-dsDNA^c^ (≥200 IU/mL) or positive anti-dsDNA autoantibody (≥30 IU/mL) Systemic Lupus Erythematosus Disease Activity Index ≥4 For 30 day prior, stable immunosuppressive therapy with steroid (0-20 mg/day) or other immunosuppressive medication such as hydroxychloroquine, chloroquine, azathioprine, methotrexate, mycophenolate mofetil, cyclosporine, belimumab, rituximab
Exclusion	Pregnancy Active lupus nephritis, myocarditis, or pericarditis Physical activity more than 2 times a week

^a^ACR: American College of Rheumatology.

^b^EULAR: European League Against Rheumatism.

^c^dsDNA: anti–double stranded DNA.

### Study Design

**Figure 1:**
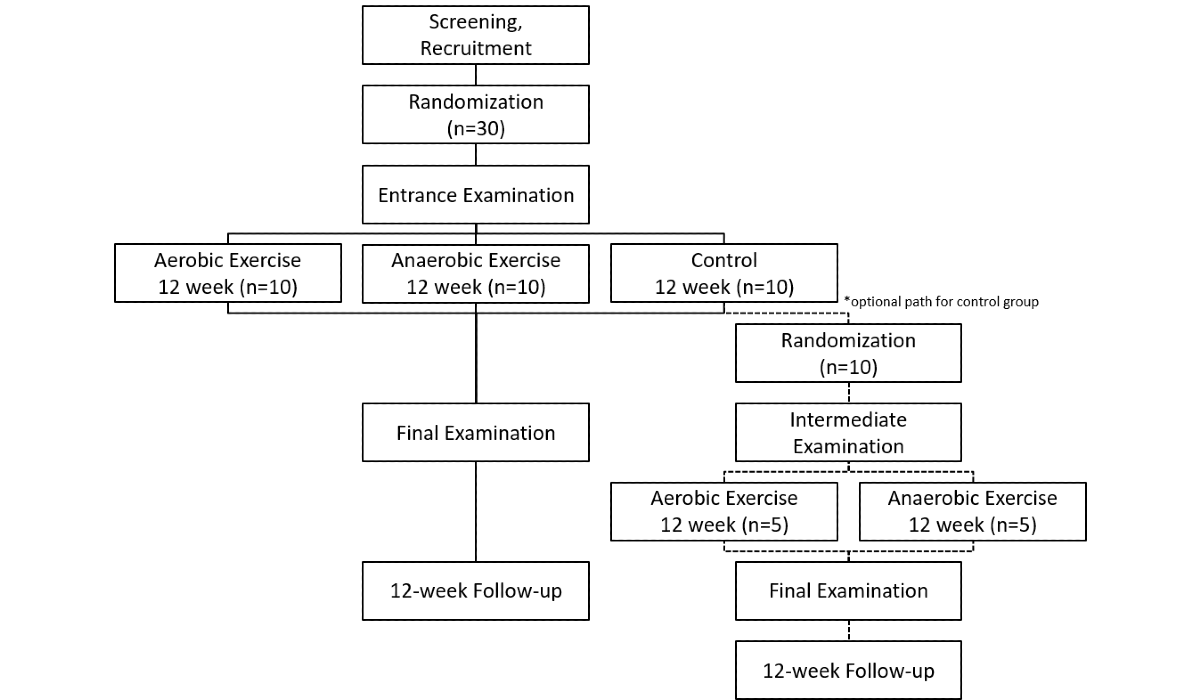
Study design.

After recruiting and screening participants (n=30), they were randomized to 3 groups: mainly aerobic training (n=10), mainly anaerobic training (n=10), and control (treatment as usual; n=10). The patients in the control group have the option to participate in one of the exercise groups after 12 weeks of treatment as usual (Figure 1). By creating 2 different exercise groups, we wished to study whether there are different effect sizes and differences in feasibility. As far as we know, no data are available on anaerobic training conditions for patients with systemic lupus erythematosus.

During the examinations and follow-up appointments, which take place in the Division of Nephrology and Rheumatology outpatient clinic of the University hospital Mainz and the Department of Sports Medicine of the University in Mainz, blood samples are taken, and questionnaires are filled out. During the outpatient visits, the patients are cared for by the study doctor and a doctoral student in sport medicine. Participants undergo preliminary examination, which includes bioelectric impedance analysis (InBody 3.0, InBody Co Ltd), a Chair-Stand test (as many complete sit-stand-sit cycles as possible, with a chair height of 43.2 cm, in 30 seconds) [23], resting electrocardiography (ECG) (AT-60; Schiller Medizintechnik GmbH), and a pulmonary function test (Bodybox 5500, Medisoft Group).

During physical fitness testing, patients undergo spiroergometry and ECG. To determine individual physical performance, participants undergo standardized stepwise exercise test on a laboratory treadmill (Saturn, H/p/cosmos Sports & Medical GmbH) until participative exhaustion, with a modified walking protocol (Table 2) that includes 14 steps of increasing velocity and partially increasing slope [28]. Absolute and relative contraindications as well as stop criteria to terminate spiroergometry were defined according to the guidelines [29]. The same physical fitness tests are repeated at the end of the 12-week period to evaluate the effect of the training program of each participation.

**Table 2:** Modified walking protocol [28].

Stage	Time, minutes	Cumulated time, minutes	Velocity, km/h	Slope, %
1	3	3	3.0	1.5
2	3	6	3.7	3.0
3	3	9	4.4	4.9
4	3	12	5.1	6.3
5	3	15	5.8	7.4
6	3	18	6.5	8.2
7	3	21	6.5	9.8
8	3	24	6.5	11.4
9	3	27	6.5	13.0
10	3	30	6.5	14.6
11	3	33	6.5	16.2
12	3	36	6.5	17.8
13	3	39	6.5	19.4
14	3	42	6.5	21.0

### Intervention

After exercise testing, patients in both intervention groups were given an individual account for the internet platform. In a personal introduction appointment after exercise testing, participants received information about the internet platform and all materials, such as the smartwatch (M430, Polar Electro Oy) and 3 different resistance bands (KG 67071, 67072, 67073, Trendy Sport GmbH & Co). The smartwatch was used to evaluate the physical strain of the patients (heart rate) and distance during exercise. Participants had the opportunity to enhance social contacts by using the smartwatch’s internal message function. Training videos for home-based resistance training could be downloaded from the platform.

Every Monday, an individualized training schedule was sent to each patient in both intervention groups. Participants are given a weekly protocol, where all physical activities during the week, including all recommended (endurance and strength) and additional activities, should be recorded. After each week, a sports therapist analyzes the training data to adapt the schedule for the following week according to participant self-reported values of pain and training load (Table 3).

**Table 3:** Weekly individual training adjustment [28].

Pain^a^		Load^a^	Adjustment
**If**			
	0-3	0-6	Increase
	0-3	7	Maintain
	0-3	8-10	Reduce
**Else if**			
	4-6	0-7	Maintain
	4-6	8-10	Reduce
**Else**			
	7-10	0-10	Reduce

^a^Borg scale 0-10.

To determine the exercise program, we used the FITT-VP (frequency, intensity, type, time, volume, and progression) principle based on American College of Sports Medicine guidelines [30]. Therefore, 3 exercise sessions were recommended, and each exercise program was be adjusted (intensity or duration) by interpreting of the training data and the rating of perceived pain and load every week. The recommendations are based on heart rate in training zones related to individual anaerobic threshold. The 12-week exercise program consists of 4 fixed mesocycles depending on the group (Table 4).

**Table 4:** Number of aerobic and anaerobic training sessions for each group.

Group and session		Mesocycle 1	Mesocycle 2	Mesocycle 3	Mesocycle 4
		Weeks 1-3	Weeks 4-6	Weeks 7-9	Weeks 10-12
**Aerobic exercise group**					
	Aerobic training sessions	3	3	3	3
	Anaerobic training sessions	0	0	0	0
**Anaerobic exercise group**					
	Aerobic training sessions	3	2	1	0
	Anaerobic training sessions	0	1	2	3

Both intervention groups undergo the same aerobic exercise program in the first mesocycle. Afterward, differently structured mesocycles in both intervention groups are used to verify the effects of aerobic or anaerobic exercise, respectively. Each training session contains endurance exercise between 20 and 50 minutes, including a 5-minute warmup, and a 5-minute cooldown, depending on the current progression stage. Every patient can scale up, scale down, or remain on different progression stages from week 1 to week 12 according to individual training adjustments (Table 4). The aerobic exercise group performs aerobic training sessions for the whole program. In the anaerobic exercise group, more intense training sessions are progressively integrated (Table 5). For the anaerobic training sessions, we use an intermittent protocol with heart rate above the individual anaerobic threshold for 2-3 minutes per interval. The progression stages in the anaerobic exercise group range from 3 intervals (1 interval of 3 minutes + 2 intervals of 2 minutes each) up to 8 intervals (8 intervals of 3 minutes each) with a 2-minute walking break between intervals, a 5-minute warmup, and a 5-minute cooldown.

To assess the effect of the intervention program, the control group (treatment as usual) will participate in voluntary exercise that is assessed using a questionnaire for habitual physical activity [31]. These participants also received a smartwatch.

We recommended that walking or running should be the main part of endurance training sessions. Moreover, we suggested performing 1 to 2 strength training session weekly or integrating specified strength training exercises into the endurance training (eg, at the end of running or walking).

We created a compilation of 10 strength exercises for major muscle groups that can be trained separately with elastic resistance bands. We recommended 3 sets with 15 repetitions per exercise each week [32]. Furthermore, the compilation includes 10 relaxation exercises, recommended for after strength training sessions.

**Table 5:** Outcome parameters.

Outcome		Description		Measurement timepoint	
**Primary outcome**					
	VO_2_ peak^a^		Using spiroergometry		Week 0, 12
**Secondary outcomes**					
	Fatigue Scale for Motor and Cognitive Functions		This scale consists of 20 items using a 5-point Likert scale, from absolutely agree to absolutely disagree) to assess cognitive fatigue (10 items) and motor fatigue (10 items). The scores for cognitive and motor fatigue are added for the sum score. A cutoff value of 43 indicates mild fatigue, whereas higher values are associated with moderate fatigue (≥53) or severe fatigue (≥63) [33].		Week 0, 12, 24
	Beck Depression Inventory		This questionnaire consists of 21 sets of statements, which are ranked in terms of severity from 0 to 3. The sum (range 0-63) indicates the severity of depression. The standardized scale is 0-8, no depression; 9-13, minimal depression; 14-19, mild depression; 20-28, moderate depression; 29-63: severe depression [34].		Week 0, 12, 24
	Systemic Lupus Erythematosus Disease Activity Index		This index consists of 24 items including clinical and laboratory variables to measure disease activity within the previous 10 days. The maximum score is 105, scores >3 indicate a mild or moderate flare, and scores ≥12 indicate a severe flare.		Week 0, 12, 24
	Disease Activity Score–28		The score indicates rheumatoid arthritis disease activity and treatment response. It is composed of 4 measures including the number of swollen or tender joints, C-reactive protein level, and patient’s health assessment. A total score is calculated using the formula. Values range from 2.0 to 10, where a higher value indicates higher disease activity. The score is a valuable tool to assess the severity of joint involvement and activity in systemic lupus erythematosus.		Week 0, 12, 24
	Work Ability Index		This self-assessment questionnaire is used to assess the work ability of the patients. The questionnaire covers 6 dimensions including current work ability, as well as past 2-year estimation among others: 7-27 points indicates poor, 28-36 points indicates moderate, 37-43 points indicates good, and 44-49 points indicates very good work ability.		Week 0, 12, 24
	Revised Cutaneous Lupus Erythematosus Disease Area and Severity Index		This scoring system includes a score to measure the activity of skin lesions and a score to measure the damage of skin lesions in patients with discoid lupus erythematosus and cutaneous lupus erythematosus. The score is used as a follow-up parameter. It has been shown that scores correlate well with the physicians and patient’s global assessment of disease activity.		Week 0, 12, 24
	Autoantibody titer		dsDNA^b^ titer (standard value ≤20 IU)		Week 0, 12, 24
	Complement level		C3c and C4 levels (standard values: C3c: 0.9-1.8 g/L; C4: 0.1-0.4 g/L)		Week 0, 12, 24
	Circulating, cell-free DNA levels		The concentration of circulating, cell-free DNA (ng/mL) is measured before during and after laboratory standardized stepwise exercise test from capillary and venous blood samples. After centrifugation of the samples, the circulating cell-free DNA is determined by a direct quantitative real-time polymerase chain reaction method from plasma without previous DNA extraction [31] Compared to healthy participants patients with systemic lupus erythematosus show higher circulating cell-free DNA plasma levels.		Week 0, 12
	Extracellular vesicles		The relative amount of extracellular vesicle subpopulations is analyzed using bead isolation and size exclusion chromatography followed by protein marker characterization.		Week 0, 12
	Lactate levels		To estimate the lactate threshold, capillary blood samples are taken from the fingertips using end-to-end capillary with a defined volume of 20 µL (sodium heparin, EKF-Diagnostics GmbH). Erythrocytes are hemolyzed in glucose/lactate hemolyzing solution (EKF-Diagnostics GmbH) before analysis using the Biosen S-Line (EKF-Diagnostics GmbH). In this study, capillary blood samples are taken at the beginning of the test (pre), after each step of treadmill walking, as well as 3 minutes after exhaustion. All samples are quantified directly after the test. To define the anaerobic lactate acid threshold or individual anaerobic threshold the Dickhuth model (baseline +1.5 mmol/L model) is used [35].		Week 0, 12
	Ventilatory threshold		Change in ventilatory threshold after 12 weeks compared to baseline.		Week 0, 12
	Muscle mass		Muscle mass will be measured in absolute mass (kilograms) including internal organs using bioelectrical impedance analysis.		Week 0, 12
	Chair-Stand test		Change of Chair-Stand test after 12 weeks compared to baseline.		Week 0, 12
	Borg scale		Ratings of perceived exertion with the Borg 15-grade scale (6-20) within the last 30 seconds of each stage of walking will be recorded [36].		Week 0, 12
	Smartwatch data		Evaluation of the physical strain and performance during the weekly training sessions measured by heart rate and distance covered during running.		Week 0-12

^a^VO2 peak: peak oxygen uptake.

^b^dsDNA: anti–double stranded DNA.

### Outcomes

#### Primary Outcome

The primary objective of this study is to examine changes in physical fitness in response to an internet-based exercise program with aerobic or anaerobic training protocols in patients with systemic lupus erythematosus. Therefore, the primary outcome is the change of VO_2_ peak (after 12 weeks in comparison to baseline).

#### Secondary Outcomes

The secondary outcome parameters are summarized in Table 5. This table also shows at what timepoints parameters are measured.

## Results

The study was registered in May 2019 (NCT03942718). Information brochures were laid out in the rheumatology outpatient clinic of the University Medical Center Mainz for the recruitment of patients. In addition, information brochures were sent by mail to patients with systemic lupus erythematosus treated in the rheumatology outpatient clinic. Out of 40 patients who contacted us, 30 patients fulfilled the inclusion criteria and were included in the study. One patient withdrew before the first performance test and before the start of the sports program due to a fracture, and 29 patients started the study. Among the 25 patients who completed the study, no serious adverse events were reported; however, 1 patient has not yet completed the study, and 3 patients withdrew from the study. One due to repeated colds, so that regular sport was not possible, another patient had a relapse of Crohn disease during the study period, and 1 patient stated that continuing to exercise was not possible due to physical strain.

## Discussion

Previous studies [20,21] indicate that exercise can lead to various benefits in patients with systemic lupus erythematosus due to an increased aerobic capacity, exercise tolerance, and quality of life as well as decreased depressive symptoms and symptoms of fatigue. These positive effects were achieved in supervised as well as unsupervised exercise programs [15,17-19,22,37,38]. In addition, it has been repeatedly discussed whether physical activity leads to an increase in lupus disease activity—an increase in autoantibodies, an increase in the consumption of complement factors, and an exacerbation of clinical symptoms such as arthralgia and myalgia. However, several recent studies [18,20] have shown that physical activity is safe in patients with systemic lupus erythematosus and that there is no increase in lupus disease activity.

Nondrug therapy, especially exercise therapy, has become an important component of long-term treatment of comorbidities of systemic lupus erythematosus in recent years. Therefore, effective and efficient exercise programs need to be studied. In this study, we focus on the feasibility of an internet-based exercise program in patients with systemic lupus erythematosus. Similar concepts have already been successfully applied in other diseases such as major depressive disorder, fatty liver disease, Barrett carcinoma, cystic fibrosis, and psychiatric disorders [28,39-41].

To our knowledge, this is the first study in which exercise treatment in systemic lupus erythematosus is supervised via the internet, which has several benefits: (1) Patients with systemic lupus erythematosus can perform their individual exercise program at a self-chosen time point and do not need to participate in a presence program. Moreover, participants can fulfil their exercise program at home, which has several logistic benefits. (2) To reduce the risk of physical over- or underload, a weekly feedback protocol, which includes the rating of perceived exertion and the heart rate during the exercise sessions, is used. Based on these data, participants receive weekly-adapted exercise prescription, which allows adjustments in accordance with FITT-VP principles throughout the program. Moreover, we ensure a moderate beginning of exercise prescription to avoid injuries or dropout based on exercise overload. Other studies found a positive association between adherence, compliance, or persistence and treatment satisfaction [42] In this study, adherence can be evaluated by completed training sessions. (3) By using the platform, participants can benefit due to a chat and forum function. (4) This allows patients to communicate quickly with the sports therapist, gain insight into their own training sessions, and have the opportunity to communicate with each other. Through this exchange, there is a possibility of mutual motivation enabling social connection and lasting training bonds among participants. By using the internet to supervise the exercise program, resources are reduced, since one sports therapist can supervise participants in parallel.

Tench et al [14] showed that patients with systemic lupus erythematosus (n=93) have reduced oxygen uptake in comparison to that of healthy controls (n=41). Furthermore, Keyser et al [43] compared 16 healthy participants with 18 patients with systemic lupus erythematosus and found significant differences in their aerobic capacity (VO_2_ peak). A meta-analysis showed an improvement of nearly 2 mL/kg/minute of oxygen uptake after exercise treatment (12-52 weeks) [21]. Wilson et al [44] assume that low aerobic capacity leads to a significant restriction in daily life. Mostly all daily activities have a range of 10.5 mL/kg/minute to 17.5 mL/kg/minute of oxygen uptake. In addition, Pinto et al [45] showed that patients with systemic lupus erythematosus have impaired aerobic capacity when compared with the aerobic capacity of controls matched by physical inactivity, age, sex, and BMI. These findings reinforce the recommendation of physical activity in systemic lupus erythematosus treatment and were recently reconfirmed by a randomized one-year physical activity program for women with systemic lupus erythematosus [46].

However, patients with systemic lupus erythematosus frequently suffer from fatigue in their daily life, so the burden of being physically active is much higher than it is for healthy patients. But when the patients are physically active, a reduction in fatigue could already be observed after a walking program of 6 weeks [22]. Thus, an individually adapted training program (based on training status and time of training) could be continuously and easily used by patients and to positively support permanent use.

Nevertheless, there are some risks in using the internet as a supervision tool. First, we presuppose that all participants have an internet device and an email account to create a user profile. Research has shown that nearly 79.5% of the population in Germany has access to an internet account [47]. Second, it could be possible that patients need the presence of a personal coach to perform the exercise sessions correctly, even to reduce the risk of injury. Therefore, precise recommendations of exercise prescription will be used and controlled. It is also possible to watch the strength exercises as videos on the internet platform. Furthermore, it already has been outlined above that systemic lupus erythematosus progress is heterogeneous which could be a critical point of this study. Patients could suffer due to health-associated problems, which could lead to an early termination of the exercise program, as they have no personal contact person on site. However, our objective is to promote a frequent contact through the platform and that the patients will not have any inhibitions and will be able to report at any time. Complaints about health problems are always passed on to the study physician so that immediate contact with the patient is possible. In this way, we hope to achieve a reduction of the risk of premature termination of the program.

A selection bias due to (1) exercise affine people and (2) internet-based motivated people could be possible. Moreover, based on etiology, it is expected that (3) mostly female participants will participate. Furthermore, (4) only participants followed by the University Medical Centre Mainz will be recruited.

This study will allow us to assess the potential for internet-based exercise program in patients with systemic lupus erythematosus by comparing our internet-based exercise program in terms of changes in levels of physical ability (VO_2_ peak, anaerobic exercise group, Chair-Stand test) in the treatment groups compared to those in the treatment as usual group. Furthermore, it will allow us to study the intensities of exercise recommendations that are feasible for patients with systemic lupus erythematosus without any disadvantages in terms of disease activity.

## Abbreviations

BMI: body mass index
ECG: electrocardiography
FITT-VP: frequency, intensity, type, time, volume, and progression
VO2 peak: peak oxygen uptake
